# Co-expression of *KLK6* and *KLK10* as prognostic factors for survival in pancreatic ductal adenocarcinoma

**DOI:** 10.1038/sj.bjc.6604717

**Published:** 2008-10-14

**Authors:** F Rückert, M Hennig, C D Petraki, D Wehrum, M Distler, A Denz, M Schröder, G Dawelbait, H Kalthoff, H-D Saeger, E P Diamandis, C Pilarsky, R Grützmann

**Affiliations:** 1 Visceral, Thoracic and Vascular Surgery, University Hospital Carl Gustav Carus, Technical University of Dresden, Fetscherstrasse 74, Dresden 01307, Germany; 2 Department of Nephropathology, Evangelismos Hospital, Phedriadon 109, Athens 11364, Greece; 3 Bioinformatics Group, Biotechnological Centre, Technical University Dresden Tatzberg 47/49, Dresden 01307, Germany; 4 Division of Molecular Oncology, Clinic for General Surgery and Thoracic Surgery, Schleswig–Holstein University Hospitals, Arnold-Heller-Str. 7, Kiel 24105, Germany; 5 Department of Pathology and Laboratory Medicine, Mount Sinai Hospital, 600 University Avenue, Toronto, ON M5G 1X5, Canada

**Keywords:** pancreatic cancer, *KLK10*, *KLK6*, DNA-microarray, microenvironment

## Abstract

Kallikreins play an important role in tumour microenvironment and as cancer biomarkers in different cancer entities. Previous studies suggested an upregulation of *KLK10* and *KLK6* in pancreatic ductal adenocarcinoma (PDAC). Therefore, we evaluated the clinicopathological role of these kallikreins and their value as biomarkers in PDAC.

Differential expression was validated by DNA-microarrays and immunohistochemistry in normal and malignant pancreatic tissues. Sera concentrations of both kallikreins were evaluated using ELISA. *In silico* analysis of possible protein interactions and gene silencing of *KLK10 in vitro* using siRNAs gave further insights in the pathomechanisms.

Gene expression analysis and immunohistochemistry demonstrated a strong expression for *KLK10* and *KLK6* in PDAC. Statistical analysis showed that co-expression of these kallikreins correlated with an R1-resection status (*P*=0.017) and worse outcome for overall survival (*P*=0.031). Multivariate analysis proofed that co-expression is an independent prognostic factor for survival (*P*=0.043). Importantly, *KLK10* knockdown in AsPC-1 cells significantly reduced cell migration, whereas computational analysis suggested interaction of *KLK6* with angiogenetic factors as an important mechanism.

Co-expression of *KLK10* and *KLK6* plays an unfavourable role in PDAC. Our results suggest that this effect is likely mediated by an interaction with the factors of the extracellular matrix and enhancement of cancer cell motility.

Pancreatic ductal adenocarcinoma (PDAC) is one of the most aggressive cancers with an incidence rate of 6.3/100 000 (Lowenfels and Maisonneuve, 2006; Jemal *et al*, 2008). It is characterised by early metastasising and aggressive, infiltrative growth along the endothelium basement membrane and neurons (Eccles and Welch, 2007). This leaves more than 85% of the patients inoperable at the time of diagnosis. Unfortunately pancreatic carcinoma also shows an unsatisfactory response to oncological treatment (Wolff *et al*, 2000). This demonstrates the need for new therapeutic approaches and also for biomarkers, which make early diagnosis possible. Recently, we (Grutzmann *et al*, 2003) and others (Iacobuzio-Donahue *et al*, 2003; Yousef *et al*, 2004) showed that human kallikrein 10 and human kallikrein 6 are among the most highly and specifically overexpressed genes in pancreatic cancer compared with normal and benign pancreas tissues.

*KLK10* and *KLK6* are members of the kallikrein family of 15 known proteases in humans, which play an emerging role in tumour microenvironment, invasion and angiogenesis (Borgono and Diamandis, 2004). Kallikreins exert this function as secreted trypsin and chymotrypsin-like proteases by degradation of the extracellular matrix, which is an important reservoir for cytokines and growth factors such as VEGF, TGF-*β* and kininogens (Borgono and Diamandis, 2004). Moreover, *KLK3*, also known as prostate-specific antigen, is of great clinical value as a serological marker in prostate cancer (Borgono and Diamandis, 2004). Other members of the kallikrein family might also have a utility in screening of malignancies, like *KLK11* and *KLK6* for ovarian cancer (Diamandis *et al*, 2003; McIntosh *et al*, 2007). This leaves kallikreins as advantageous candidate genes for diagnosis and therapy in pancreatic cancer. Therefore, the aim of this study was to evaluate the clinicopathological role of these kallikreins and their value as biomarkers in PDAC.

*KLK10*, also known as the normal epithelial cell-specific 1 is one of the newly identified members of the kallikrein family. Its role in carcinogenesis is unsolved as it is downregulated in some tumours such as breast cancer and acute lymphoblastic leukaemia, whereas overexpressed in ovarian, prostate or renal cancer (Luo *et al*, 2003; Petraki *et al*, 2003, 2006; Zhang *et al*, 2006). Unfortunately, the function of *KLK10* protein remains poorly documented, neither the activators nor the substrates for *KLK10* are actually known (Zhang *et al*, 2006). *KLK6*, or protease M, is highly expressed in several malignancies like ovarian, breast, colon or gastric cancer. It is correlated with lymphatic invasion and poor prognosis in gastric cancer (Yousef *et al*, 2004; Nagahara *et al*, 2005). *KLK6* might exert this effect by the degradation of matrix proteins and thereby the augmentation of cancer cell motility and proliferation (Ghosh *et al*, 2004).

In this study, we show that kallikrein 10 and 6 demonstrate a strong protein expression in pancreatic carcinoma and are associated with poor patient prognosis and R1 resection status and thereby might contribute to the aggressive character of this malignancy.

The results indicate that this effect is most likely mediated by the interaction of *KLK6* with factors of the extracellular matrix and the enhancement of cancer cell motility by *KLK10*.

## Materials and methods

### Patients and demographic data

For immunohistochemical analysis we used samples from 54 patients, operated from July 1996 until December 2003. None of the patients received adjuvant chemotherapy prior to operation. The eligibility criterion was a histologically confirmed PDAC. A positive microscopic resection margin (R1) was operationally defined as at least one cancer cell within 1 mm of any surface of the resected specimen. We included patients with the finding of metastases to the intra-aortocaval lymph nodes. Previous reports have shown that these metastases should not exclude patients in good condition from oncologic resection of the PDAC (Shrikhande *et al*, 2007). Relevant patient characteristics are summarised in Table 1.

For serum-ELISA several panels of sera were selected from 130 healthy donors, eight patients with benign tumours, 34 with inflammatory diseases and 28 patients with malignant diseases (Table 2). All sera were from patients treated at the University Hospital Dresden, Germany and University Hospital Schleswig–Holstein, Germany. All patients had given informed consent.

### Construction of a virtual subarray and bioinformatic analysis

For the construction of the virtual subarray we used data obtained from the U133 A/B Affymetrix GeneChip using extracted RNA from microdissected tissue as described earlier (Pilarsky *et al*, 2008).

The Cel Files obtained from the Affymetrix MAS 5.0 software were used for further analysis. The files were loaded into dCHIP 1.3 (www.dchip.org) then normalised, and expression values as well as absolute calls were calculated using the PM/MM model (Grutzmann *et al*, 2003). We scored genes as differentially expressed if they displayed a fold change >2. To identify signature genes we used the method described elsewhere (Grutzmann *et al*, 2004). Datasets are accessible (Arrayexpress E-MEXP-1121 and E-MEXP-950).

### Immunohistochemistry study and evaluation

Immunostaining was carried out on paraffin-embedded tissue sections by streptavidin—biotin–peroxidase complex method using polyclonal antibodies for *KLK6* and *KLK10* (1 : 150). The ductal epithelium and the Langerhans' islets served as positive controls for both kallikreins (Petraki *et al*, 2001, 2002a, 2003). Negative controls were performed for all studied tissues by omitting the primary antibody (KLK6 and KLK10) or by replacing it by non-immune serum (dilution 1 : 500) (see Supplementary Data 1).

All sections were examined by one observer (CDP) blinded to both clinical and pathological data. Protein expression in PDACs was quantified using a visual grading system with a range between 0 and 2, based on the intensity and the proportion of positive tumour cells on the studied section: **0**=no immunoexpression or weak staining in any proportion of the cancerous tissue, or moderate expression in </=5% of the cancerous tissue, 1=moderate staining in 5–50% of the cancerous tissue, 2=moderate staining in >50% of the cancerous tissue or strong staining in any proportion of the cancerous tissue. According to staining intensity, cancers were classified as low, medium or high expressing tumours. Coexpression of both *KLK*s was defined as strong if the sample showed strong expression for both *KLK*s (2/2) and/or at least showed moderate expression for one *KLK* (2/1). *KLK6* and *10* immunoexpression were also screened in the normal pancreatic parenchyma (acinar, ductal and endocrine cells) and in the ampulla of Vater region of the small intestine.

### Patient serum selection and ELISA for kallikrein measurement in serum

ELISA-type immunofluorometric procedures developed in-house were used to measure *KLK6* and *10* levels in these sera. Assays used in this study were of the ‘sandwich’ type with the primary antibody used for capture and the secondary one for detection. Monoclonal–monoclonal combinations were used in this study. All ELISAs were tested negative for cross-reactivity against other kallikreins. Assay precision within the dynamic range was <10%. These assays were standardised with recombinant proteins produced in yeast or mammalian expression systems. More details about the kallikrein ELISA have recently been published (Shaw and Diamandis, 2007).

### Protein interaction prediction

To evaluate the interactions we queried databases with known protein–protein interactions such as NetPro (www.molecularconnections.com), SCOPPI (www.scoppi.org) and HPRD (www.hprd.org) and compared them to our data. To find possible novel interactions we used structure- and sequence-based prediction of protein interactions as described earlier (Altschul *et al*, 1997; Mishra *et al*, 2006; Dawelbait *et al*, 2007).

### Cell culture and transfection conditions

The AsPC-1 cell line (ATCC Number CRL-1682), established from malignant ascites of a 62-year-old female caucasian, was used for this study. Cells were grown in RPMI-1640 (Invitrogen, Karlsruhe, Germany) with 2 mM L-glutamine, 1 mM sodium pyruvate, 4.5 g l^−1^ glucose and 10% FCS in a humidified atmosphere containing 5% CO_2_ at 37°C. AsPC-1 cells (50 000) in media with 1% FCS were transfected with 600 ng of siRNA using oligofectamine (Invitrogen GmbH, Karlsruhe, Germany). Target sense sequences that effectively mediated silencing were as follows: KLK10.1 (UACAUGUCCUGGAUCAAUA) and KLK10.2 (UGACGUGCCUACCUCUUAG) (all from MWG Biotech, Ebersberg, Germany). Knockdown was confirmed by RT–PCR and western blot. siRNA against the green fluorescent protein (GGCUACGUCCAGGAGCGCACC) served as a negative control. For RT–PCR the following cell lines were used: Capan-1 (ATCC No. HTB-79), Capan-2 (ATCC No. HTB-80), MiaPaCa-2 (CRL-1420), Panc1 (ECACC No. 7092802), BXPC3 (CRL-1687) and Panc 89, Colo357, PancTUI, PT45P1 (all from Professor H Kalthoff, Kiel, Germany, (Sipos *et al*, 2003)).

### Reverse transcription–polymerase chain reaction

Using RNeasy Mini Kit (Qiagen, Hilden, Germany) we isolated total RNA and subjected 500 ng to cDNA synthesis using random primer and SuperScript II (Invitrogen GmbH, Karlsruhe, Germany). Of the synthesised cDNA 2% were used for quantitative RT–PCR. Analysis was performed using an ABI PRISM 5700 Sequence Detection System (Applied Biosystems, Weiterstadt, Germany). The genes were amplified with the Power SybrGreen PCR Master Mix according to the manufacturer's instructions. Gene expression was quantified by the comparative *C*_t_-Method, normalising *C*_t_-values to a housekeeping gene (*β*-actin) and calculating the relative expression values. Each experiment was repeated two times in duplicate. The following primers were used: *β*-Actin: Actb 1498 f (AAGCCACCC-CACTTCTCTCTAA) and actb-1570R (AATGCTATCACCTCCCCTGTGT), *KLK10*: KLK10_f1 ex (GTCCTGGTGGACCAGAGTTG) and KLK10_r1_ex (GAGCTG-CTCTCCCTGAAGAA), *KLK6*: KLK6_f1 (GTG TGC TGG GGA TGA GAA GT) and KLK6_r1 (GGG ATG TTA CCC CAT GAC AC) (all from MWG Biotech, Ebersberg, Germany).

### Western blotting

Cells were washed and lysed with LDS sample buffer (Invitrogen, Karlsruhe, Germany). Proteins were electrophoresed under reducing conditions on 4–12% acrylamide gels (Invitrogen, Karlsruhe, Germany) and then transferred to a nitrocellulose membrane (Hybond ECL, GE Healthcare, Munich, Germany). To block nonspecific binding, the membrane was incubated overnight in PBS with 0.1% Tween 20 (T-PBS) containing 5% BSA at 4°C. Subsequently, the membrane was incubated for 1 h with the antibody against *KLK10* (1 : 1000, see (Petraki *et al*, 2001, 2002a, 2003)) or *β*-actin loading control (1 : 5000, no. ab6276, Abcam, Cambridge, UK) in T-PBS and 5% BSA. After washing in T-PBS, protein on the membrane was visualised using the ECL detection kit (GE Healthcare, Munich, Germany) with a peroxidase-labelled anti-mouse antibody for *β*-Actin (1 : 25000, no. NIF 825, Amersham Pharmacia, Amersham, United Kingdom) and peroxidase-labelled anti-rabbit antibody for *KLK10* (1 : 5000, no. NIF 824, Amersham Pharmacia, Amersham, United Kingdom) as per manufacturer's instructions. Protein expression was measured by AIDA evaluation software (Raytest, Straubenhardt, Germany) as the ratio of KLK10-staining intensity to actin-staining intensity.

### Boyden chamber assay

Invasion *in vitro* was measured in Boyden chamber assay (no. 353097, BD Falcon, Heidelberg Germany). The PET membrane had a pore size of 8 μm with a pore density of 1.0 × 10^5^ cm^−2^. Cells were transfected using the above-mentioned protocol and incubated for 48 h. Cells were then trypsinised, counted and cell suspensions of the two groups (5 × 10^5^ cells per 250 *μ*l) were transferred in 1% FCS medium onto the membrane. Then the chambers were put in 24-well plates containing 10% FCS medium and cultured for 72 h. Cells infiltrated through the reconstituted basement membrane and appeared on the outer surfaces. By HE staining the number of the cells was counted microscopically. Migration assays were repeated three times.

### Statistical analysis

*P*-values were assigned using *χ*^2^ (Pearson) for the cross tables, the log-rank test (Mantel–Cox) for the survival univariate analysis by the Kaplan–Meier test, and the Cox regression analysis for multivariate survival analysis. For statistical analysis ‘SPSS 13.0’ for Windows was used.

## Results

### Virtual subarray

**Figure 1:**
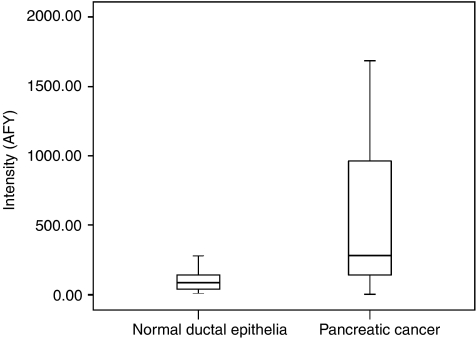
Results of the GeneChip analysis. *KLK10* showed a marked upregulation in pancreatic cancer samples compared with normal individuals (*P*=0.009).

Gene expression profiles of 19 patients with PDAC and normal ductal cells from 13 individuals were generated (Pilarsky *et al*, 2008). We then constructed a virtual subarray to identify gene expression changes of the 15 kallikreins. The nine probe sets identified with the virtual subarray analysis represented six differentially expressed genes (Table 3). Of these, *KLK10* was overexpressed whereas *KLK*3, 12, 13 and 15 were downregulated in PDAC compared with microdissected normal ductal cells. Upregulation of *KLK10* was strong (Figure 1) compared with normal individuals (*P*=0.009). We also decided to consider *KLK6* for further evaluation because upregulation was found in PDAC by other groups (Iacobuzio-Donahue *et al*, 2003; Yousef *et al*, 2004).

### Immunohistochemical expression of KLK6 and KLK10

In the endocrine pancreas, the immunohistochemistry for *KLK6* and *KLK10* showed strong staining in the endocrine cells of the Langerhans' islets and in scattered endocrine cells in connection with pancreatic ducts and acinar cells.

**Figure 2:**
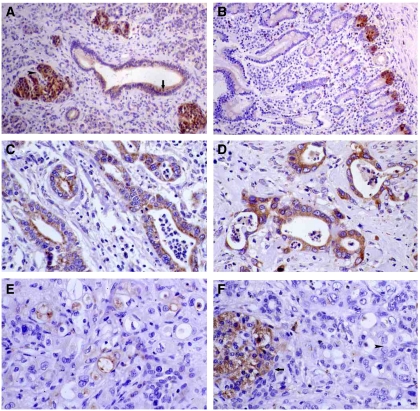
Immunohistochemical staining of PDAC samples. Moderate *KLK6* immunoexpression in pancreatic ducts (arrow) and strong expression in Langerhans' islets (arrowhead) no staining in acini ( × 100) (**A**). Strong *KLK10* immunoexpression in the crypts of the intestinal epithelium of the ampulla of Vater ( × 100) (**B**). Strong *KLK6* immunoexpression in pancreatic adenocarcinomas ( × 200) (**C** and **D**). Moderate *KLK10* immunoexpression in pancreatic adenocarcinomas ( × 200) (**E**). Strong *KLK10* immunoexpression in Langerhans' islets (arrow), absence of expression in pancreatic adenocarcinoma (arrowhead) ( × 200) (**F**).

The exocrine part of the pancreas displayed a cytoplasmic expression in the small intercalated pancreatic ducts, the intra- and inter-lobular pancreatic ducts, the main pancreatic duct and the common bile duct. Staining was absent in the acinar cells (Figure 2A and E). In the region of the ampulla of Vater in the small intestine, a strong cytoplasmic, mostly supranuclear immunoexpression was observed in the epithelium of the intestinal crypts. The absorptive cells in the surface villous epithelium showed a moderate cytoplasmic and brush border expression, whereas goblet cells were mostly negative (Figure 2B).

The staining for *KLK6* in primary PDAC showed a moderate to strong expression in 91.5% of the cases, whereas it was only 64.4% for *KLK10*.

**Figure 3:**
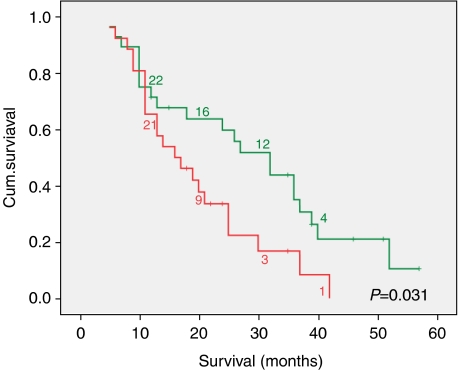
The survival curve shows a lower medium survival time of 20 months (15.0–24.0) in the subgroup of patients with strong *KLK6* and *KLK10* co-expression compared with patients without/weak expression of these kallikreins (29 months (22.8–35.8)) (*P*=0.031).

*KLK6* showed a diffuse cytoplasmic immunostaining in the cancerous epithelium, whereas *KLK10* mostly showed a patchy expression, often with luminal pattern (Figure 2). Analysis of immunohistochemistry revealed that patients with strong *KLK6* and *KLK10* co-expression had significant lower medium survival time of 20 months (15.0–24.0) compared with patients without/weak expression of these kallikreins (29 months (22.8–35.8)) (*P*=0.031) (Figure 3). Neither *KLK10* (*P*=0.259) nor *KLK6* (*P*=0.452) expression could be correlated with survival alone. We could further associate high *KLK6* and *KLK10* immunoreactivity with R1-resection status (*P*=0.017) (Table 1).

Cox regression analysis identified *KLK10* and *KLK6* co-expression as an independent prognostic factor with a statistically significant relationship to survival in PDAC (*P*=0.043, RR 2.002) (Table 4).

### KLK10 and KLK6 serum concentration

Using ELISA immunoassays developed in-house, we tested *KLK6* and *KLK10* serum levels in patients with malignant, inflammatory and benign diseases; additionally there was a panel of healthy patients.

There was no significant correlation of *KLK6* and *KLK10* serum levels with survival of the patients. Also, there was no statistical significance between healthy donors and patients with PDAC nor between different localisations of inflammatory, benign and malignant diseases of the pancreatico-biliary tract (Table 2).

### Protein interaction prediction

To find potential interaction partners for *KLK6* and *10*, which might explain poor survival, we used two different computational methods: the structure- and the sequence-based protein interaction prediction. By means of these methods we could identify four potential interaction partners for *KLK6*: α-1 antiproteinase, AT III, pigment epithelium-derived factor and synuclein (Table 3). However, no additional interaction partner for KLK10 other than the already described could be identified (Dawelbait *et al*, 2007).

### Expression of KLK10 and KLK6 in pancreatic cancer cell lines

**Figure 4:**
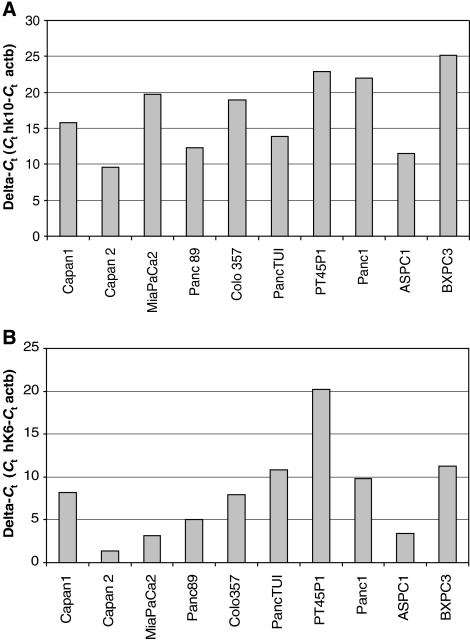
A qRT–PCR of different established pancreatic cancer cells lines showed Capan-2 and AsPC-1 with relevant *KLK10*-expression (**A**). *KLK6*-expression was high in nearly all measured cell lines (**B**).

To evaluate expression of *KLK10* and *KLK6* in established cell lines we conducted a qRT–PCR. Capan-2, Panc89 and AsPC-1 cell lines displayed the highest *KLK10* expression. For *KLK6* the cell lines Capan-2, Mia PaCa-2 and AsPC-1 showed a good expression. Like in immunohistochemistry of native tumour, *KLK6* expression in cell lines was more distinct than that of *KLK10* (Figure 4).

### Gene silencing of KLK10 in AsPC-1 cells

To elucidate the relevance of dysregulations of *KLK10* for carcinogenesis, we established a siRNA assay. For this purpose, we used the human pancreatic cancer cell line AsPC-1. This cell line showed detectable *KLK10*-expression as well as *KLK6*-expression (Figure 4).

**Figure 5:**
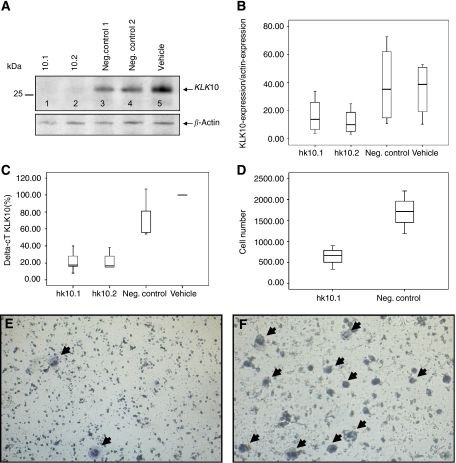
AsPC-1 cells were transfected with two *KLK10*-sequences, KLK10.1 and KLK10.2. Transfection resulted in a strong downregulation of *KLK10* protein synthesis in the western blot. Cell lysates of KLK10.1- and KLK10.2-transfected cells showed nearly no staining (lanes 1 and 2) compared with the controls (lanes 3–5) (**A**). Statistical analysis showed a statistical downregulation of the transfected group compared with the control group (*P*=0.025) (**B**). The same holds true for gene expression in RT–PCR (*P*<0.001) (**C**). The Boyden chamber migration assay: Invasion *in vitro* was measured as described in ‘Materials and methods’. Statistical analysis showed that KLK10.1-transfected cells had a significant decrease in cell motility compared with the controls (*P*=0.05) ( × 200) (**D**). Only few KLK10.1-transfected AsPC-1 cells migrated through the membrane (arrow) (**E**). The control group displayed normal cell migration (arrows) (**F**).

Cells were transfected with two *KLK10* sequences, KLK10.1 and KLK10.2. Untreated cells and cells transfected with unspecific siRNA served as a negative control. As shown in Figure 5 the expression of *KLK10* was strongly inhibited compared with controls in RT–PCR (*P*<0.001). The same holds true for protein synthesis in western blot (*P*=0.025). *β*-actin levels showing equal quantities of protein were loaded (Figure 5); all experiments were repeated four times. Because transfection with KLK10.1 showed the best inhibition of protein synthesis we chose this siRNA for our further studies. To examine off-target gene-silencing in our cells, we used the BLAST database (www.ncbi.nlm.nih.gov/BLAST) to search the human genome for any complementary sequences with at least 11 contiguous nucleotides matching the RNAi sites we used. No gene appeared to have sequence similarity. Transfection of AsPC-1 cells with KLK10.2 siRNA did not change the *KLK6* expression levels as measured qRT–PCR (see Supplementary Data 1).

### Migration assay

AsPC-1 cells transfected with KLK10.1 siRNA showed a significant decrease in cell motility compared with the GFP-transfected cells. Although only 636 cells (±285) could be isolated from the membrane in *KLK10* knockout cells, it was 1189 (±508) in the control group (*P*=0.05) (Figure 5).

## Discussion

*KLK10* and *KLK6* are among the most highly and specifically overexpressed genes in pancreatic cancer compared with normal and benign pancreas tissues (Grutzmann *et al*, 2003; Iacobuzio-Donahue *et al*, 2003; Yousef *et al*, 2004).

Our study confirmed a marked overexpression of *KLK10* in PDAC by means of a virtual subarray. Immunohistochemistry in native tumour tissue could prove not only an intense expression for *KLK10* in 64.4% of the malignant cells, but also for *KLK6* in 91.5%. Both proteins were located in the cytoplasm, from where they are likely to be secreted (Borgono *et al*, 2004).

Co-expression of different kallikreins, similar to the situation found in our study, was already reported in skin and different glands. In these tissues the kallikreins can act independently, but also together as part of proteolytic cascades (Petraki *et al*, 2002b; Borgono and Diamandis, 2004). The latter seems to be an important mechanism in pancreatic cancer, because expression of *KLK10* itself could not be associated with poor survival in PDAC, whereas the co-expression of both kallikreins was significantly associated with poor survival and an R1-resection status, which is an indirect sign for infiltrative and aggressive growth. In multivariate analysis, the co-expression of *KLK10* and *KLK6* was also an independent risk factor for survival.

It is most interesting, in which ways kallikreins affect cellular signalling and thereby contribute to cancer progression. It was already reported that kallikreins influence communication between malignant cells and their environment by degradation of extracellular matrix and thereby facilitate tumour invasion and metastasis (Borgono and Diamandis, 2004). In case of *KLK6*, degradation of fibrinogen, laminin, fibronectin and collagen types I and IV are documented (Bernett *et al*, 2002; Magklara *et al*, 2003). This cleavage of fractions of the ECM might be of specific importance in pancreatic carcinoma, which is a tumour type with a very high content of stromal tissue (Pilarsky *et al*, 2008). In contrast, functional data on *KLK10* are very limited. Although Zhang *et al* (2006) suggested, that *KLK10* was not even an active protease, it was stated in the same report that neither the protein relevant for conversion of *KLK10* into its active form nor the physiological substrates for *KLK10* are known. So, the importance of *KLK10* in tumour progression remains unclear.

It therefore seems crucial to further pinpoint some of the components, which might be responsible for the pathophysiological effect of *KLK10*. To find possible interaction partners for both kallikreins we used the *in silico* method of protein interaction prediction. By means of this method we could identify four potential interaction partners for *KLK6*. Although α-1 antiproteinase seems to be an inhibitor for *KLK6* action, the interaction with AT III shows a branching between kallikreins and blood coagulation cascade, as already reported earlier (Borgono *et al*, 2004). Another interaction partner was pigment epithelium-derived factor (PEDF), which is the major circulating inhibitor of plasmin. With this interaction, PEDF is linked to the plasminogen activator/plasmin system, which is one of the main protease systems involved in tumour cell invasion and metastasis (Hayashido *et al*, 2007). Only recently PEDF was also identified as a key inhibitor of stromal vasculature in the mural pancreas. *In vivo* androgen ablation increased PEDF in human cancer biopsies, which might also be an indirect sign for the interaction of the androgen-responsive kallikrein family and PEDF (Doll *et al*, 2003). The interaction between *KLK6* and PEDF seems highly significant and studies are under way which will further evaluate this topic. Another interaction partner we found is synuclein, which integrates presynaptic signalling and membrane trafficking in neurons. The high expression of *KLK6* might thereby play an important role in various pathologic processes of pancreatic cancer.

Although a specific interaction partner for *KLK10* could not be found, our study implies that it might have a role in the pathophysiology of PDAC. To ascertain the contribution of *KLK10* to pancreatic cancer microenvironment, we used siRNA-mediated gene-silencing (Hammond *et al*, 2000). AsPC-1 cells, which inherently express high levels of *KLK10* mRNA, were transfected with specific siRNA. We could not observe an effect on proliferation or apoptosis in *KLK10*-silenced cells (data not shown). But *KLK10*-suppressed clones had markedly reduced cell motility in the Boyden chamber assay. The number of cells migrating through the membrane along an FCS-gradient dropped more than 50%. This is highly significant, as *KLK6* was also shown to reduce cell motility (Ghosh *et al*, 2004).

Although high expression in pancreatic carcinoma indicates that *KLK6* and *10* could be promising tumour markers, we could not assess the use of *KLK6* and *KLK10* as serum biomarkers in PDAC. The serum levels of both proteins showed no significant differences between patients with PDAC and healthy donors. In addition, the serum concentrations were not able to predict the localisation of malignant lesions in the pancreatico-biliary tract. This circumstance can be because of the mainly local action of the kallikreins or fast degradation. Probably future studies including more patients can prove a use for *KLK6* or *KLK10* as tumour biomarkers in PDAC.

In conclusion, this study shows that *KLK10* and *KLK6* co-expression has an unfavourable influence on the survival in patients with PDAC and was significantly associated with R1 resection status. This effect might be mediated by direct or indirect interaction of the two kallikreins. The pathophysiological mechanisms are most likely degradation of the extracellular matrix and interaction with angiogenic factors by *KLK6*, whereas *KLK10* augments cell motility. However, our findings suggest a high complexity of interactions between the kallikreins, which leaves it difficult to generally make statements about properties of single kallikreins.

It seems very promising to find out more about the physiological role of *KLK10*. Consequently, it might be possible to use inhibitors of kallikreins to disrupt interactions between the tumour and its environment and thereby reduce disease progression in patients with pancreatic cancer.

## Figures and Tables

**Table 1:** Clinicopathological variables and KLK10 and KLK6 co-expression of the tumour cohort (*n*=54)

	**All cases**	**Co-expression**		
**Characteristics**	***n* (%)**	**None/low**	**Strong**	***P*-value**
Age at diagnosis (years) 60.5 (range, 31–76)				
				
*Gender*				
Male	24 (44.4)	12	12	0.808
Female	30 (55.6)	16	14	
				
*Localisation*				
Head of pancreas	47 (87.0)	26	21	0.186
Tail of pancreas	7 (13.0)	2	5	
				
*Tumour stage*				
pT1	6 (11.1)	5	1	0.391
pT2	13 (24.1)	6	7	
pT3	32 (59.3)	16	16	
pT4	3 (5.5)	1	2	
				
*Nodal status*				
N0	22 (40.7)	12	10	0.565
N1	31 (57.4)	15	16	
N2	1 (1.9)	1	0	
				
*Interaortocaval metastasis*				
Positive	7 (13.0)	4	3	0.764
Negative	47 (87.0)	24	23	
				
*Grade*				
G1	4 (7.4)	4	0	0.096
G2	38 (70.4)	17	22	
G3	12 (22.2)	7	5	
				
*Residual tumour status*				
R_0_	41 (75.9)	25	16	0.017
R_1_	13 (24.1)	3	10	
				
*Perineural invasion*				
Yes	19 (35.2)	11	8	0.513
No	35 (64.8)	17	18	
				
*Perilymphatic invasion*				
Yes	24 (44.4)	11	13	0.429
No	30 (55.6)	17	13	

Patients with strong immunohistochemical co-expression of *KLK6* and *KLK10* showed a significant correlation with R1-Resection status (*P*=0.017).

**Table 2:** Results of the serum ELISA showed no statistical significance between diseases nor between different localisations

	**Median**		**Mean**		
**Diagnosis (Localisation)**	***KLK6***	***KLK10***	***KLK6***	***KLK10***	***P* (log-rank)**
Benign Tumours (*n*=4)	12.4	2.1	10.3±7.8	1.4±1.8	n.s.
*Inflammatory diseases*					
Bile duct (*n*=1)	12.9	1.3			
Gallbladder (*n*=4)	13.1	2.1	14±2.2	2.3±0.67	
Pancreas (*n*=12)	10	2.0	7.7±5.7	2.4±0.79	
					
*Malignant tumours*					
Bile duct (*n*=3)	8.1	1.3	7.2±4.7	1.2±0.31	
Gallbladder (*n*=3)	5.7	1.0	4.5±2.0	1.2±0.42	
Pancreas (*n*=8)	9.7	1.6	9.3±4.6	1.5±0.95	
Normal (*n*=65)	9.5	1.8	9.0±3.6	1.7±0.85	

**Table 3:** (A): Results of the GeneChip analysis. The upregulated genes (fold-change >2, *P*<5%) are listed in the upper part, the downregulated genes in the lower part of the table; (B): Sequence-based and structure-based protein interaction prediction showed four possible interaction partners for *KLK6*

**Probe set**	**HGNC symbol**	**Fold change**	***P*-value**	
A				
*Upregulated genes*				
KLK10@209792_s_at	KLK10	5.2	< 0.01	
KLK10@215808_at	KLK10	1.6	0.01	
				
*Downregulated genes*				
KLK13@216670_at	KLK13	−1.7	0.01	
KLK3@204583_x_at	KLK3	−6.6	0.03	
KLK12@233586_s_at	KLK12	−9.4	< 0.01	
KLK12@234316_x_at	KLK12	−10.1	< 0.01	
KLK1@216699_s_at	KLK1	−10.2	< 0.01	
KLK12@220782_x_at	KLK12	−10.5	< 0.01	
KLK15@221462_x_at	KLK15	−15.3	< 0.01	
				
B				
**Protein 1**	**Predicted partner**	**Also known as**	**Function**	**Compartment**
*KLK6*	SERPINA3	*α*-1 antiproteinase	DNA-binding, endopeptidase inhibitor activity, inflammatory response	Extracellular, intracellular, nucleus
	SERPINC1	AT III	Blood coagulation	Extracellular region
	SERPINF2	Pigment epithelium-derived factor	Endopeptidase inhibitor activity, protein-binding, acute phase response	Extracellular region
	SNCA	Synuclein	Anti-apoptosis, central nervous system, development, protein binding	Cytoplasm
*KLK10*	No interaction partner found			

**Table 4:** Cox regression model, including conventional variables and co-expression of *KLK6* and *KLK10* in all cases (*n*=54)

	**Relative risk**	**95% CI**	***P*-value**
Co-expression *KLK*6/*KLK10*	2.022	1.021–4.006	0.043
Resection status	0.571	0.247–1.321	0.190
Grading	1.964	1.030–3.746	0.040
pT stage	0.498	0.310–0.801	0.004
Nodal status	1.814	0.937–3.513	0.077
Metastases	0.545	0.177–1.679	0.290

Co-expression is a strong independent prognostic factor for survival in patients with PDAC (*P*=0.043).
