# Supplementary material for: Co-expression of KLK6 and KLK10 as prognostic factors for survival in pancreatic ductal adenocarcinoma
Source: Br J Cancer. 2008 Oct 14;99(9):1484–92. doi: 10.1038/sj.bjc.6604717 (PMC2579692; doi:10.1038/sj.bjc.6604717)
Supplement: Supplementary Data 1 [file 6604717x1.pdf]

Supplemental Data 1.

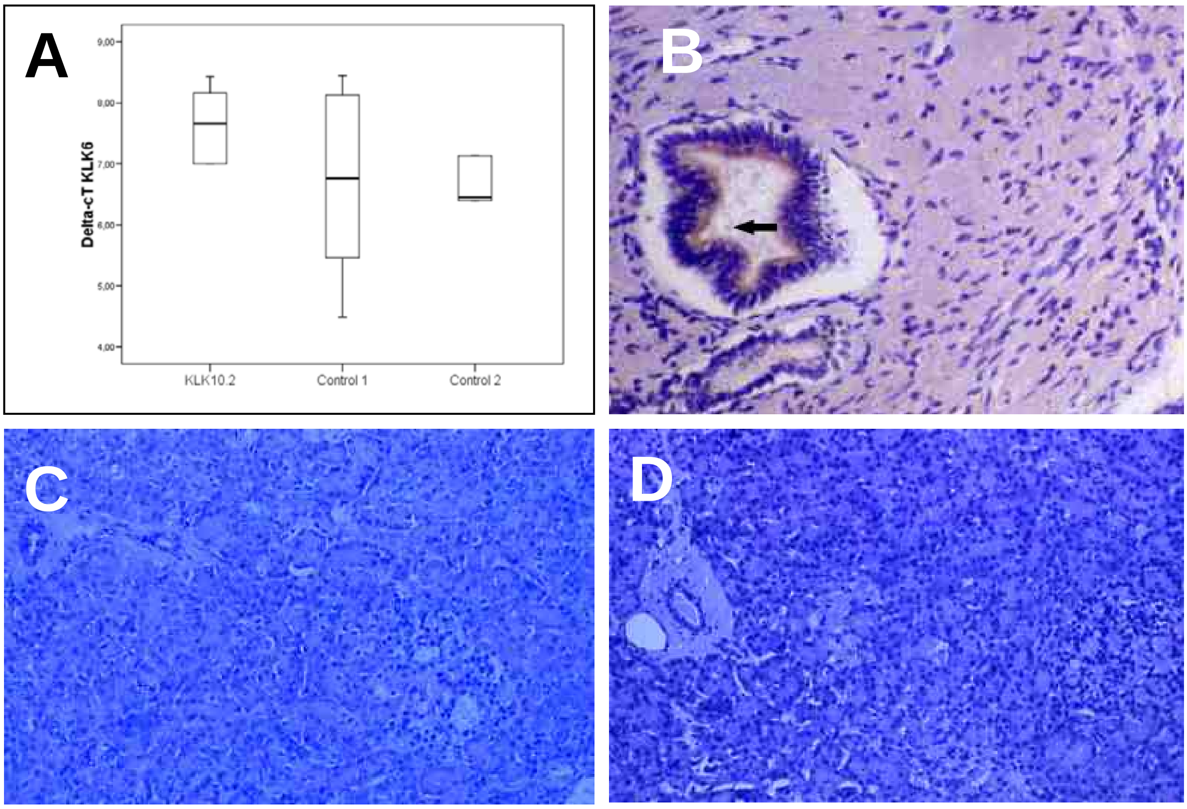

Supplemental Data1: Expression of *KLK*6 in the transfected cell line. No difference in expression of KLK6 was observed between cells transfected with KLK10.2 and control 1 (p=0.466) resp. control 2 (p=0.977) (**A**). Moderate hK10 immunoexpression in a pancreatic duct (arrow) (x100) (**B**). Negative control without the first Ab in normal pancreatic tissue showed no staining (KLK6 or KLK10) (x100) (**C**). No staining in normal pancreas, by replacing the primary antibody (KLK6 or KLK10) by non-immune serum (x100) (**D**)
